# Metabolomics-Based Evaluation of Crop Quality Changes as a Consequence of Climate Change

**DOI:** 10.3390/metabo11070461

**Published:** 2021-07-16

**Authors:** Helena Romero, Delphine M. Pott, José G. Vallarino, Sonia Osorio

**Affiliations:** 1 Instituto de Hortofruticultura Subtropical y Mediterránea “La Mayora”, Departamento de Biología Molecular y Bioquímica, Universidad de Málaga-Consejo Superior de Investigaciones Científicas, Campus de Teatinos, 29071 Málaga, Spain; hromero@uma.es (H.R.); dpott@uma.es (D.M.P.); 2 Departamento de Biología Molecular y Bioquímica, Universidad de Málaga, 29071 Malaga, Spain; vallarino@uma.es

**Keywords:** fruit, climate change, metabolites, metabolomics, abiotic stress

## Abstract

Fruit composition determines the fruit quality and, consequently, consumer acceptance. As fruit quality can be modified by environmental conditions, it will be impacted by future alterations produced by global warming. Therefore, agricultural activities will be influenced by the changes in climatological conditions in cultivable areas, which could have a high socioeconomic impact if fruit production and quality decline. Currently, different stresses are being applied to several cultivated species to evaluate their impact on fruit metabolism and plant performance. With the use of metabolomic tools, these changes can be precisely measured, allowing us to determine changes in the patterns of individual compounds. As these changes depend on both the stress severity and the specific species involved and even on the specific cultivar, individual analysis must be conducted. To date, the most-studied crops have mainly been crops that are widely cultivated and have a high socioeconomic impact. In the near future, with the development of these metabolomic strategies, their implementation will be extended to other species, which will allow the adaptation of cultivation conditions and the development of varieties with high adaptability to climatological changes.

## 1. Introduction

Climate change is a challenge that we are already facing and will need to be overcome in the near future according to predictions. This change, which is mainly influenced by the emission of greenhouse gases by anthropogenic activities, will not be the same all around the globe; thus, how climatic conditions change will depend on the region and season [1,2]. Among others, a general increase in the temperature, solar irradiance, and changing precipitation patterns as well as a higher frequency of droughts are expected [3,4]. In particular, these key climatic change factors are gaining considerable attention in different forums due to the effects on agriculture, which cause important socio-economic losses.

These abiotic stresses affect plant growth, development, and metabolic processes, thus, reducing the yield and quality of crop plants. The evolution of the future climate is uncertain, and the current cultivation strategies will need to be adapted to new environmental conditions. Furthermore, certain cultivars will not be suitable for the areas where they are currently being cultivated, and if climatic conditions drastically change, they will need to be relocated [5] to prevent the crop yield or fruit quality from deteriorating. To avoid this and subsequent dramatic economic losses [6], tolerant and adaptable cultivars with acceptable production and quality must be explored [5,7,8].

On the other hand, the impacts that climate change can have on crops are genotype-dependent; therefore, the response against the same stress can differ [2,9,10,11]. Moreover, depending on the plant developmental stage, as well as the stress severity and its duration, the outcomes of plant growth and reproduction differ [12,13]. Even though abiotic stresses can have negative effects on crops, in some cases, a moderate stress can produce improvements in fruit quality with little impact on the fruit yield [14,15]. Considering that the plant growth response to key climate change factors has been more widely studied, this review is focused on fruit quality research that is still more unexplored [13].

The term “quality” has been defined in several ways depending on customer groups, such as breeders, producers, and consumers. Fruit quality is determined by a combination of traits, including the flavour, colour, and texture. The metabolic composition has a direct impact on these characteristics [8]. Additionally, certain metabolites have been shown to have health beneficial properties, showing potential bioactivities, such as anti-oxidant, anti-cancer, anti-microbial, and anti-neurodegenerative characteristics [16,17]. Metabolites can be subdivided into products of the primary and secondary metabolisms [18]. 

In fruits, the central primary metabolites, including sugars, sugar alcohols, organic acids, amino acids, and fatty acids—key compounds of fruit taste—act as precursors of secondary metabolites, such as volatile, phenylpropanoid, and terpenoid compounds, responsible for the colour, aroma, and nutritional characteristics of fruits [18,19,20,21,22,23,24,25]. Particularly, the main pigments, flavonoids and carotenoids, have an important role in fruit appearance and, therefore, in its consumer quality perception [26,27,28]. In addition, these compounds have been reported to be involved in plant defences against biotic and abiotic stresses and also have specific health benefits due to their antioxidant activities [23,24]. Therefore, the changes produced in the metabolism will not only impact plant development but also modulate fruit quality and consequently influence consumer acceptance and the fruit’s nutritional value [11,29].

Metabolomic technologies allow a comprehensive qualitative and quantitative analysis of the numerous metabolites in biological system. Thus, it is a powerful tool to (i) identify putative metabolic biomarkers affecting fruit quality by different genotypes, growing conditions, and environmental changes; and (ii) investigate based on metabolic changes, how the metabolic processes are modulated under different stresses.

The physico-chemical characteristics and concentrations of metabolites are variable; therefore, sensitive and selective techniques for their accurate identification and quantification are required [30]. Among all available techniques, the most commonly used in metabolomic studies related to changes due to climatic conditions is chromatography coupled with mass spectrometry (MS) (Table 1).

Gas chromatography (GC) has been widely applied to characterize compounds related to the central metabolism as well as volatiles with high reproducibility [31]. GC-MS technologies have a high resolution, which allows the robust quantification of a few hundred metabolites within a single extract. These techniques have achieved a high sensitivity and are mainly applicable to volatiles and non-volatile polar metabolites, mainly metabolites from the primary metabolism (amino acids, sugars, organic acids, and fatty acids), through derivatization by methylation or trimethylsilylation.

Here, metabolites can be identified by comparing the retention index and fragment patterns with those of standard compounds. GC-MS-based metabolomic platforms have some other advantages, like having (i) a large number of well-developed databases for metabolite identification, and (ii) stable protocols for machine setup, maintenance, and usage [32]. The main limitation of GC-MS technologies is the restriction to analyse compounds that are volatile or made volatile through chemical derivatization [33,34]. 

Liquid chromatography (LC) coupled to MS is a versatile technology used to analyse a wider range of metabolites without a prior derivatization step, mainly compounds from the secondary metabolism [33,34]. Additionally, many LC-MS instruments are equipped with MS/MS, providing useful information for predicting partial metabolite structures. However, in contrast to GC-MS, peak annotation is more difficult since metabolite database are not as rich as GC-MS libraries [32].

## 2. Heat Stress

Increasing global temperatures are one of the main stresses plants have to deal with as a consequence of climate change. For this century, an increase in temperatures is expected, accompanied by severe heatwaves [20,53,55]. In plants, high temperature exposure induces important developmental and physiological disorders, which are highly dependent on the severity of the stress and its duration [12,13,20]. Moreover, fruit composition is strongly affected by the environment, for which it is expected that global warming will induce dramatic changes in metabolite contents [30,55]. 

**Figure 1:**
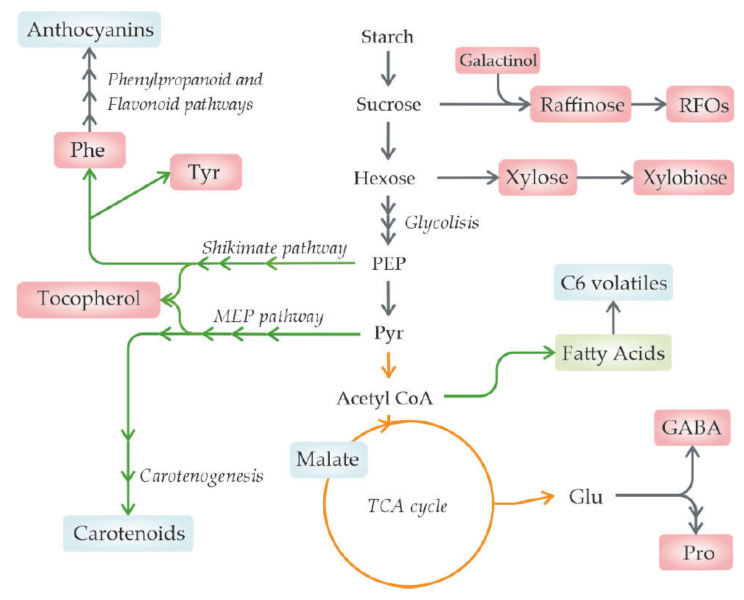
General effects of heat stress in fruit metabolism. Red: increase; blue: decrease; and green: changes in its patterns. Arrows: grey: cytosol; orange: mitochondria; and green: plastids. RFOs: raffinose family of oligosaccharides.

A typical signature response is to synthetize protective molecules, such as osmoprotectants and antioxidants, such as reactive oxygen species (ROS) to maintain cell and membrane integrity and deal with the oxidative stress produced by heat stress [55] (Figure 1). Therefore, understanding the metabolic changes that heat stress produces in fruits will allow the generation of cultivars and cultivation strategies suitable for the future environment without the loss of beneficial properties [40].

### 2.1. Impacts on Primary Metabolism

The next paragraphs are focused on the impacts of heat stress on the production and accumulation of primary metabolites in plant edible parts and the resulting consequences on fruit quality attributes. 

#### 2.1.1. Sugars

Sugars are the main structural components of plants in addition to providing energy for growth and reproduction. Sucrose is the principal sugar transported from photosynthetic tissues to sink organs, such as fruits [85]. Once in the fruit, sucrose may be hydrolysed into hexose (fructose and glucose) or may serve as a building block for carbohydrate polymer synthesis, such as starch or cellulose [86]. Sucrose, fructose, and glucose are generally the most abundant sugars present in ripe fruits, and these directly impact their taste. 

Moreover, the ripening process is driven by crosstalk between sugars, particularly sucrose, and phytohormones, making carbohydrates key molecules in fruit development and maturation [86]. Furthermore, sugar concentration is also related to the synthesis of secondary metabolites, as it promotes the accumulation of the anthocyanins and carotenoids involved in fruit appearance [18,87].

Grapevines (*Vitis vinifera* L.) have a high socio-economic impact around the world, with numerous hectares dedicated to grape and wine production [40]. Thus, in this crop, sugar concentration has a high relevance because it not only affects the fruit flavour and its acceptance but also determines the final alcohol content in wines [88,89,90].

In grapes, it has been described that an excessive increase in temperature can stop sugar accumulation and consequently stop the ripening process [88]. In addition, it is also described that the final sugar content is dependent on duration of heat stress. In grape cell cultures of cv. Gamay Red, under severe stress, an increase in the content of different monosaccharides, such as fructose 6-phosphate and glucose 6-phosphate, was observed [47]. However, when the stress lasted longer, the content of these monosaccharides decreased, while xylose and xylobiose increased, suggesting that these sugars may take part in cell protection, as they are involved in lignification [47,91].

The developmental stage of the fruit when stress is applied can also influence sugar accumulation, as has been observed in grapes [38] and tomato [13]. In grapevines under heat stress at early developmental stages, when sugar accumulation is still low, high temperatures have triggered a delay in ripening and sugar accumulation, but when the stress was applied at later stages, this accumulation was accelerated [38]. Sugar accumulation was also accelerated in grapes from the cultivar Pinot Meunier at higher temperatures, although the final concentration did not change between lower (22/12 °C day/night) and higher temperatures (30/20 °C day/night) [40]. 

This surprising observation could be explained by the fact that the expression of most primary metabolic genes remains unchanged with temperature. On the other hand, in the tomato (*Solanum lycopersicum* L.) cv. Ailsa Craig, under a simulated heatwave, an increase in sucrose content was observed when the stress was applied at early ripening stages, concomitant with the previously described sensitivity of the sucrose metabolism to elevated temperatures in tomato reproductive organs and young fruits, while no significant differences were observed in the more mature stages [13]. The observed metabolite accumulation was also consistent with the transcriptomic analysis, showing a downregulation of the genes involved in sucrose degradation at early stages [13]. 

An increase in sucrose, glucose, and fructose was also observed in the tomato cv. “Money-maker” when a relatively moderate increase in temperature (29.4 °C) was applied [39]. These increase in the main sugars in tomato fruits resulted in an increase in starch degradation [92], and could result in an acceleration in fruit maturation [39]. However, with lower temperatures (24 °C) applied to different black currant (*Ribes nigrum* L.) cultivars, they all showed less sugar content than berries cultivated at lower temperatures [10]. Therefore, the observed outcome depends on the studied fruit species and the magnitude of the stress.

Additionally, other soluble sugars are found at lower concentrations, playing key roles in signalling, development, and abiotic stress tolerance [86,93]. In particular, one of the mechanisms plants deploy to cope with the damage that heat stress can produce in cells is the production of osmoregulators [24,94]. In particular, the accumulation of sugars from the raffinose family oligosaccharides synthesized from sucrose and galactinol appears to be closely related to plant responses to environmental stresses, such as desiccation, oxidative, cold, or heat stresses [95]. 

In leaves of *Coffea arabica* L. under heat stress, the raffinose and stachyose contents increased; therefore, an increase in temperatures could trigger this protective mechanism [9]. Under heat stress, grapes from the cultivar Cabernet Sauvignon increased in the galactinol concentration, the first step in the production of raffinose family oligosaccharides, although no changes in the raffinose or stachyose content were detected [36]. On the other hand, in Gamay Red cell cultures, an increase in the oligosaccharides of the raffinose family was observed [47].

#### 2.1.2. Organic Acids

Together with sugars, organic acids are the main contributors to fruit taste, conferring acid savour [10]. Citric and malic acids are commonly the most abundant organic acids in fruits [10,52], decreasing the sucrose and fructose sweetness, respectively [52]. Regarding the observed changes in these compounds in fruits exposed to different heat treatments, they generally increase in citric acid content and decrease in malic acid, as was reported in black currants [10], strawberries [37], and tomatoes [39]. However, the magnitude of the observed changes can depend on the studied cultivar [10,37]. In the strawberry (*Fragaria* x *ananassa* Duch) cv. Fortuna, the increase in citric acid was more notable when compared with the other cultivars studied, while malic acid decreased in the cultivar Festival but fluctuated in the others [37]. 

Additionally, the rate of the changes can vary depending on the developmental stage that the fruits were in when they suffered the stress, as has been observed in grapevines, with a faster decrease in malic acid when the stress was applied at advanced developmental stages [40]. In grapevine cell cultures, the observed effect was the opposite of that previously described, showing an increase in malic acid and a decrease in citric acid. This response could be due to the stress being applied for a period of 24 h [47]. Other acids found in fruits include tartaric, shikimic, and fumaric acids [10], although no significant changes have been observed in tartaric acid in grapevines under heat stress [40].

Apart from the organoleptic attributes that they confer to fruits, organic acids are also involved in the fruit ripening process and, consequently, are important for fruit stability [96]. During this process, organic acids tend to decrease, as has been observed in strawberry [50] and tropical fruits [51]. Perhaps the observed increase in sugars and a decrease in malic acid in tomato were not accompanied by differences in the contents at maturation under heat stress (29.4 °C) [39]. Malic acid breakdown and sugar accumulation are two processes linked to fruit ripening [40]. When grapevines were exposed to higher temperatures, the malic acid breakdown was faster and started simultaneously with sugar accumulation. 

This observation was further corroborated by transcriptomic analyses, which found a reduced glycolytic flux and an increased TCA anaplerosis, which was unfavourable to malic acid accumulation in heated fruits. However, under cooler temperatures, an uncoupling of malic acid breakdown and sugar accumulation was observed, with the malic acid decrease delayed until the sugar concentration increased to 0.4–0.5 M, accompanied by a delay in fruit ripening [40]. This striking desynchronization may be the result of a more favourable carbon status of the plant under cool conditions, which prevents malic acid consumption and neoglucogenesis. Taken together, these observations suggest that heat stress may hamper fruit metabolic efficiency [40].

#### 2.1.3. Amino Acids

Amino acids are needed for protein synthesis; however, several attributes have also been given to these compounds [97,98]. Most importantly, amino acids can act as precursors of several secondary metabolites in plants [18]; in this way, alterations in these compounds can consequently produce changes in the secondary metabolism.

Furthermore, the amino acid composition is especially relevant in grapes, as its content can affect wine characteristics, since yeasts can use them to produce both desirable and undesirable products [99]. Generally, an increase in different amino acids is observed under heat stress in different grapevine cultivars, although the pattern of which amino acid increases differs between cultivars [25,53]. 

In Cabernet Sauvignon, the total increase in amino acids is mainly due to the increase in arginine, threonine, tyrosine, phenylalanine, cysteine, lysine, and GABA [53], while in Tempranillo grapevines, arginine, proline, threonine, and glutamine are responsible for this increase [25]. Remarkably, the increase in arginine can lead to the production of biogenic amines, commonly found in wines, with harmful effects in humans [99]. It is worth highlighting the increase in GABA, as this metabolite acts as a signalling molecule and controls the carbon–nitrogen balance [100,101].

Under heat stress, an increase in phenylalanine, a precursor of many secondary metabolites, and a substrate of phenylalanine ammonia-lyase (*PAL*), the key enzyme in the phenylpropanoid pathway [102], was observed both in black currants [35] and in grapes from the cultivar Cabernet Sauvignon [53]. In fact, phenylalanine accumulation is most likely the result of the strong repression of *PAL* genes under heat stress [53]. Additionally, an increase in tryptophan, a precursor of phytohormone indole-3-acetic acid, which is involved in the repression of ripening [103], was observed in black currant, underlying one of the possible mechanisms by which heat stress negatively impacts fruit maturation [35].

#### 2.1.4. Fatty Acids

Dietary fatty acids play an essential role in maintaining health status, as it has been established that an unbalanced intake and proportion in the body are correlated with a general increase in the risk of different diseases. Saturated fatty acids are linked to a proinflammatory situation, with an increase in ROS production and an association with insulin resistance, among other effects [104]. On the other hand, monounsaturated and polyunsaturated fatty acids, such as oleic acid and omega-3 fatty acids, respectively, have the opposite effect, inducing beneficial effects as they decrease insulin resistance and prevent inflammation [104]. In fact, the replacement of saturated fatty acids with polyunsaturated fatty acids has shown a reduction in the risk of coronary diseases, mainly by a reduction in LDL and thrombogenesis [23].

Olives (*Olea europaea* L.) represent a crop with high economic importance, and their fatty acid composition principally determines the obtention of a high-quality oil [105]. Oleic acid is the major fatty acid found in all olive cultivars and is highly associated with benefits due to its high antioxidant activity [105,106]. Unfortunately, the fatty acid content is strongly affected by heat stress [107], changing the olive’s properties and oil characteristics. In fact, decreases in olive oil concentration are considered a marker for heat exposure, as they are linearly proportional to the duration of the stress [56].

Overall, a decrease in the oleic acid content has been observed in olives from cultivars Arauco and Zelmati grown at higher temperatures, possibly as a consequence of an increased activity of oleic acid desaturase in warm climates [24,56,57,108]. However, while Arauco mesocarps have shown a decrease in oleic acid with increasing temperature, being higher when the stress was applied earlier in development, in the seeds, an increase in oleic acid was observed when stress was applied at earlier stages of development, when oil accumulates, and in later stages, oleic acid concentration was not affected by heat [57]. In contrast, saturated and polyunsaturated fatty acids tend to increase with higher temperatures, exceeding the limit that is established for high-quality fruits [24]. 

Palmitic and linolenic acids were found to increase with higher temperatures in the cultivar Zemalti [24], and an increase in these acids was observed in Arauco when the stress was applied for a longer period of time (four months), but their contents were not affected when the stress was shorter (one month), with the exception of linoleic acid, whose content decreased when the stress was applied at earlier stages [56]. Both mesocarps and seeds at earlier stages have shown an increase in palmitic and linoleic acids with higher temperatures, although, at 24 °C, the linoleic acid content in seeds started to decrease [57]. Therefore, a decrease in the oil quality at warmer temperatures was observed under heat stress conditions [24].

In tomatoes, lipid remodelling was found to occur when heat stress was applied with an increase in storage lipid triacylglycerol and a decrease in the unsaturation of membrane lipids [13]. In addition to its role in preserving lipid integrity under high temperatures, lipid remodelling may be interpreted as a defence mechanism against oxidative stress generated under heat. Indeed, decreased levels of membrane polyunsaturated lipids, which are easily susceptible to oxidation, might serve as a strategy to minimize lipid oxidation [13]. Lipidomic results obtained by GC-MS were supported by a transcriptomic analysis, which showed higher expression levels of genes involved in triacylglycerol synthesis and a reduction in genes that encode fatty acid desaturases [13].

### 2.2. Impacts on Secondary Metabolism

The main secondary metabolites produced by plants belong to polyphenol and terpenoid groups, which are responsible, together with volatile compounds, for fruit organoleptic and healthy beneficial properties [18].

#### 2.2.1. Polyphenols

Polyphenols are a heterogeneous group of secondary metabolites derived from the shikimate and phenylpropanoid pathways that are involved in plant relationships with the environment under different stresses [18,47], and it is expected that their contents will be influenced by the abiotic stresses associated with climate change.

##### Anthocyanins

Anthocyanins are water-soluble pigments that are responsible for red, blue, and purple colours in plants [28,109]; for example, they are responsible for red grape and wine colours [25]. Additionally, they are involved in different processes related to the interactions of the plant with the environment [28]. The variation of these compound contents in fruits has been observed to be genotype- and developmental stage-dependent. In grapes, different responses to heat stress have been observed. Furthermore, the results in cv. Sangiovese grapes have demonstrated that heat stress induces decoupling between anthocyanin and sugar accumulation, confirming previous studies regarding vegetative tissues. 

Indeed, anthocyanin accumulation was strongly impaired by heat stress, while the rate of sugar accumulation remained unaffected [110]. A decrease in the total anthocyanin content was also observed when warmer temperatures were applied to the cultivar Cabernet Sauvignon at advanced phenological stages [53], although delphinidin, cyanidin, petunidin, and peonidin contents were reduced in all stages [53]. A reduction in anthocyanins was also observed in a clone of the grape cultivar Tempranillo, which may be explained, at least partially, by a deregulation (up- or downregulation depending on the developmental stage) of genes involved in anthocyanin stabilization and/or degradation [25].

In particular, Movahed et al. [110] described the role of the peroxidase Prx31 induced under heat stress, which may be involved in anthocyanin degradation. Although there is a general reduction in anthocyanins under heat stress, an increase in certain derivatives was observed in different cultivars [25,53]. For example, in the cultivar Cabernet Sauvignon, the content of malvidin-3-*O*-glucoside, which is the most abundant in this fruit, was unaffected under heat stress, while its acylated form increased when the stress was applied to green berries [53]. In the cultivar Tempranillo, an increase in 3-acetyl-glucosides (malvidin and petunidin) was observed as a consequence of the regulation of flavonoid 3′-hydroxylase (*F3*′*H*), flavonoid 3′,5′-hydroxylase (*F3′5*′*H*), and *O*-methyltransferase. Interestingly, methoxylation has been shown to increase anthocyanin stability to heat [110].

In addition to the genotype and developmental stage, the severity of the stress can modulate the anthocyanin content. Therefore, in black currant plants, when the temperature was increased from 12 to 18 degrees Celsius, an increase in the most abundant anthocyanins in this fruit, delphinidin-3-*O*-rutinoside and cyanidin-3-*O*-rutinoside, was observed. However, when the temperature was increased to 24 degrees, the content of these compounds decreased [35]. This effect could be explained by the downregulation of genes involved in anthocyanin biosynthesis, such as chalcone synthase [111], or because these compounds were degraded under severe stress conditions [112].

The anthocyanin concentration can also vary depending on how many days have passed since the application of the treatment. In postharvest plums (*Prunus salicina* Lindl.) under two different storage temperatures, the anthocyanin concentration increased in both peel and flesh. Nevertheless, while at day 5 after treatment, the total anthocyanin concentration was higher at the highest temperature essayed (35 °C); 9 days after the stress treatment, the concentration was higher at the lowest temperature applied (20 °C) [28]. This observed effect could be explained by the transcriptional and enzyme activity results obtained in this study. 

In both treatments, an increase in the expression of genes that encode enzymes related to anthocyanin biosynthesis and in their activity was observed; however, the increase was faster in the fruits under the higher temperatures in the first few days, while in the following days, they were upregulated at the lowest temperature [28]. Furthermore, the higher production of ROS at 35 °C could also explain the anthocyanin decrease after 9 days; indeed, ROS led to increased peroxidase activity, which possibly reduces hydrogen peroxide, using anthocyanin as a substrate and yielding procatechuic acid [28,113]. Monitoring the procatechuic acid concentration, Niu et al. [28] concluded that the enzymatic degradation of anthocyanins induced by hydrogen peroxide was more severe after 9 days at 35 °C.

##### Flavonols

Flavonols determine, in conjunction with anthocyanins, the fruit colour and contribute to fruit bitterness [25]. They provide photoprotection to the plants; thus, it is expected that stresses, such as heat or irradiance could alter their content and profile. Indeed, a decrease in different quercetin derivatives has been observed after increasing the temperature in black currant fruits [35], while an increase in flavonols was observed in a clone of *V. vinifera* cv. Tempranillo under this stress; this increase may be one of the reasons why this clone is more tolerant to high temperatures [25]. 

In other clones of this cultivar, no significant changes in the total flavonol content were observed; however, their profile was changed, with a noticeable decrease in myrecitin-3-*O*-glucoside, which is the most abundant flavonol in this fruit species [25]. Quercetin derivative increases under elevated temperature should be more closely monitored in different fruit species, as studies have associated this metabolite with a protective role in the cardiovascular system [25].

##### Flavanols

Flavanols are related to a bitter flavour and astringency [114], with those in grape peels associated with agreeable wine astringency [115]. An increase in these compounds, specifically epigallocatechin and quercetin-3-*O*-glucoside, was found in a *V. vinifera* L. cv. Gamay Red cell culture under severe stress during the first few hours of heat treatment; however, when the stress lasted longer, their content was diminished [47]. Once again, this observed decline could be due to the downregulation of phenylalanine ammonia-lyase gene, which is involved in the first steps in the phenylpropanoid pathway under prolonged severe heat stress [47].

#### 2.2.2. Terpenoids

Terpenoids are a group of compounds derived from the mevalonate and methylerythritol phosphate pathways, many of which are related to fruit organoleptic and nutraceutical properties [18,116]. Within this group are carotenoids that are responsible for the red, yellow, and orange colours of fruits, such as tomatoes or carrots [116,117], and they have antioxidant activity, thus, being beneficial for both plants and humans [27].

Tomatoes are one of the main fruits that provide carotenoids in the diet [118]. Due to their importance, different assays have been performed to identify the effect that heat stress could have on carotenogenesis in these fruits. A decrease in the carotenoid content was observed when a general increase in temperature [27,64] and a simulated heatwave [13] was applied to fruits at different developmental stages. This observed reduction was more noticeable when the stresses were applied at advanced ripening stages [13,27], while no changes were observed when warmer temperatures were applied during early developmental stages, suggesting an adaptation to the stress [27]. 

This general decrease in carotenoid content could be explained by the downregulation of the key regulating gene in the pathway, phytoene synthase, under high temperatures [13,118]. Furthermore, enzymes involved in carotenogenesis have been shown to be post-translationally controlled by a series of proteases whose activity is significantly increased under heat stress conditions [13].

In addition, not all carotenoids are equally affected by stress. A general reduction is caused by a decrease in some classes of carotenoids, such as phytoene or lycopene, while others, such as beta-carotene, remain unaltered [13,27,64]. This decrease in all carotenes, except for beta-carotene (provitamin A), can be explained by the activation of beta-cyclase, which converts lycopene to beta-carotene when temperature increases [64,119]. A similar effect was observed by Gautier et al. [64], with a decrease in lycopene and its precursors phytoene and phytofluene at the highest temperature applied, while beta-carotene remained unaltered when it was compared with an increase in carotenoids at the lowest essayed temperature. 

However, at intermediate temperatures, the beta-carotene content decreased, while lycopene was unaffected [64]. These metabolomic results are consistent with the transcriptomic analysis simultaneously performed by Almeida et al. [13]. They noticed a downregulation of genes responsible for the first steps of this process, such as phytoene synthase, and an upregulation of transcription factors that repress this process in stressed fruits [13]. Additionally, no changes in beta-carotene were observed in tomatoes when the total carotene content increased [39]. 

In this case, using cv. Money-maker, an increase in the carotenoid content was observed at the higher temperature assay. This can likely be explained by the fact that this temperature was close to the optimal conditions, indicating that a slight increase in the cultivation temperature can stimulate the accumulation of these compounds, while even warmer temperatures led to a decrease in these compounds [39].

#### 2.2.3. Vitamin C or Ascorbate

The main interest in vitamin C intake is not only because of its high antioxidant activity but also because it is a cofactor in many human enzymes [120]. Due to its high antioxidant activity in plants, this molecule plays an important role in different processes, including ROS scavenging [27], whose production likely increases under high temperatures. In strawberries, where ascorbic acid is responsible for nearly 10% of its antioxidant activity [121], no significant changes were observed in fruits harvested in different months although they had been exposed to different temperatures before harvest [37]. 

With applied heat stress, a decrease in vitamin C has been observed in black currants [10,35] and tomatoes [27]. However, the observed effects in tomatoes depended on the developmental stage of the fruits when the stress was applied, showing normal levels of this vitamin when the stress was applied at earlier developmental stages, which suggests a plant adaptation to the stress [27]. Additionally, it was noticeable that an increase in vitamin C occurred in tomatoes when plants were under warmer temperatures during the flowering period, likely due to a decrease in the sink/source ratio produced by flower abortion [27].

When the content of this compound is measured, it must be taken into account that it can be found in an oxidized (dehydroascorbic acid) or in a reduced form, with the latter being the active form enabling plants to resist oxidative stress [122]. An increase in mainly the active form was observed in tomatoes, cv. Money-maker, at the highest temperature essayed (29.4 °C) [39]. The contradictory effects observed between the two tomato cultivars, an increase in this compound in cv. Money-maker, and a decrease in cv. Velasco could be because what it is considered the highest temperature essayed by Ruiz-Nieves et al. [39] (29.4 °C) was, in fact, the optimal temperature for the synthesis of this compound [39], being higher in the other study (32 °C) [27].

#### 2.2.4. Vitamin E or Tocopherol

Vitamin E content is determined by the different forms of tocopherol, which present different contributions to its activity [120]; its antioxidant activity is particularly relevant against lipid peroxidation in both plants and animals [123]. Completely different from what has been observed in other compounds, in tomatoes (cv. Ailsa craig), the vitamin E levels increased in heat-stressed plants compared with non-stressed plants, regardless of the developmental stage of the fruits when the stress was applied [13]. In this case, vitamin E content could be used as a marker in plants that have experienced stress [124]. In this study, no association was found between gene expression and tocopherol concentration, whose concentration could be increased by a redirection of carotenoid precursors to tocopherol synthesis [13].

#### 2.2.5. Volatile Compounds

Volatile compounds take part in fruit aroma, determining consumer acceptance [125]. In tomatoes, there are more than 400 detectable volatile compounds; however, only 16 positively contribute to fruit flavour. In tomatoes under heat stress at two different developmental stages, a decrease in C6 volatiles derived from C18:3 fatty acids was observed, likely due to the unavailability of these plastidial fatty acids produced by heat [13]. On the other hand, C18:2-derived volatiles were reduced only at early ripening stages. 

In advanced stages, C5 volatiles increased, as *LoxC*, involved in C6- and C5-volatile generation [126], was downregulated only in earlier stages [13]. Additionally, in advanced ripening stages, a decrease in lycopene-derived volatiles was observed, which correlates with the lower lycopene content found in heat-stressed plants [13]. Unlike other compounds also essayed in this study that recovered once the stress had ended, the volatile compound content did not return to normal levels, implying the high sensitivity of the tomato fruit volatilome to heat [13].

## 3. Drought Stress

Concurrently with alterations in temperature, climate change will also trigger variations in current precipitation patterns, modifying not only the precipitation levels but also changing the typical periods where rainfall occurs, causing periods of drought stress and flooding events [63,127,128,129,130,131]. Moreover, these changes will likely be accompanied by an increase in water demand as the global population grows [81]. Typically, drought stress leads to a diminution of crop yield [132], although stress effects depend on the severity of the drought and the developmental stage of the plant when it suffers stress [132]. 

**Figure 2:**
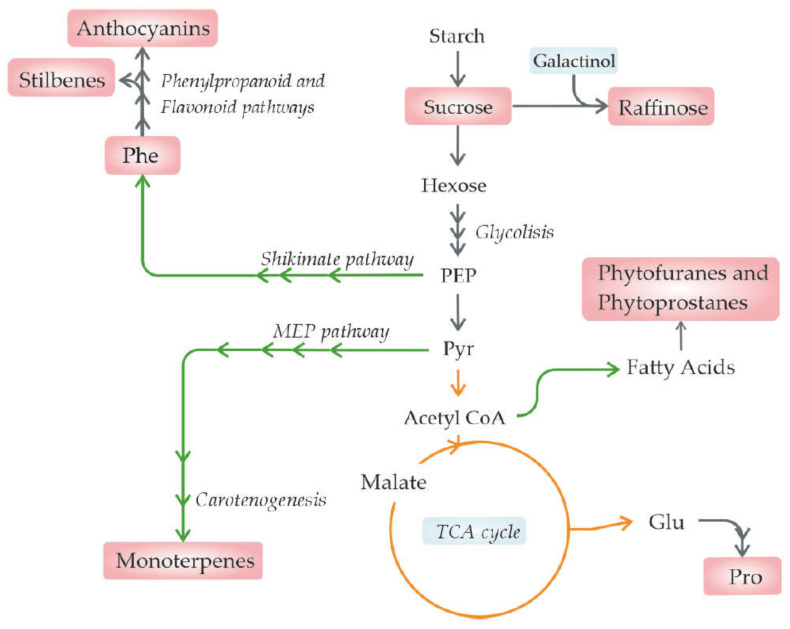
General effects of drought stress in fruit metabolism. Red: increase; and blue: decrease. Arrows: grey: cytosol; orange: mitochondria; and green: plastids.

Indeed, drought generally results in a decrease in the net photosynthesis, leading to a reduction in primary metabolite supply in fruits [42]. Furthermore, the relationship between phytohormone abscisic acid (ABA) and drought stress has been well studied [14,133,134]. In turn, as ABA participates in fruit ripening, it is expected that its induction under drought or salt stress will affect the fruit metabolic composition, in particular the sugar and anthocyanin content [14,86,135] (Figure 2). 

For these reasons, several irrigation strategies have been essayed to obtain high-quality fruits without a great loss in crop yield while optimizing the water use [62,81,136]. As the response to abiotic stress is commonly species- and cultivar-dependent, the stress impacts on fruit metabolism must be monitored individually to obtain the best performance [81].

### 3.1. Impacts on Primary Metabolism

#### 3.1.1. Sugars

Commonly, an accumulation of sugars is observed under water stress, as reported in fruits, such as kiwis [49], tomatoes [41,42], strawberries [14,46], and grapes [45,48,49]. Notably, research has described that a water deficit significantly reduces fruit weight and, thus, indirectly causes an increase in the soluble solid content [41]. Furthermore, this increase in sugars can be caused by a decrease in its utilization to obtain energy, as fruits are not growing, or due to an increase in reserve mobilization, increasing the starch degradation in stressed plants [49]. 

The individual sugars that increased depended on the studied cultivars; sucrose, glucose, and fructose increased in Shiraz grapes [48], while only sucrose accumulated in Cabernet Sauvignon grapes [45], and sucrose and fructose increased in kiwifruits during development, whereas only sucrose increased at harvest [49]. Furthermore, alterations in the sugar content disappeared when the stress was removed [49]. Sugar storage, represented by starch accumulation, increased in kiwifruit [49] and in the tomato Cervil [42] and Money-maker cultivars [137]. 

Additionally, supplying potassium under water stress was demonstrated to improve sugar accumulation in tomatoes [41]. Therefore, we can suggest that a water deficit leads to an increase in fruit quality by increasing its sweetness [41]. On the other hand, a decrease in sugars was reported in different cultivars of chilies [43], likely due to a decrease in photosynthetic activity with simultaneous sugar consumption and a redistribution of sugars to other plant organs that takes place when the stress lasts longer to maintain plant survival [43,138]. 

Additionally, osmoprotective sugars, such as raffinose, increased in Shiraz grapes at harvest, accompanied by a decrease in the precursor galactinol [48]. Interestingly, Hochberg et al. [48] found an association between grape hydraulic behaviour in response to water deficit and berry skin metabolic remodelling, which was particularly pronounced in the primary metabolism. When comparing metabolite signatures of Shiraz and Cabernet Sauvignon berries under water deficit, they observed significantly higher levels of stress-related metabolites in the first cultivar.

#### 3.1.2. Organic Acids

As pH is mainly determined by the total acid content, a decrease in organic acids is related to an increase in pH [45]. Under drought stress, a decrease in the total acid content at later stages of fruit development was observed in two different grape cultivars [45,48], although it was more pronounced in the Shiraz cultivar than in Cabernet Sauvignon, concomitant with the aforementioned increased stress signature in the first cultivar [48]. The diminished content of organic acids can be attributed to a decrease in TCA intermediates, suggestive of the compromised activity of the TCA cycle under drought stress, which is consistent with what has been observed in vegetative tissues [139]. 

Regarding individual organic acid concentrations, citric acid, which is commonly the most abundant organic acid found in fruits, decreased under deficit irrigation in Cabernet Sauvignon grapes [45] and in the chili *Capsicuum annum* var. Chili-AS Rot and *Capsicuum chinense* Jacq. var. Naga Morich [43]. On the other hand, malic acid also decreased under irrigation treatment in Cabernet Sauvignon grapes [45] but increased in the two chili species [43]. 

These two organic acids remained unaltered in mango (*Magnifera indica* cv. Lirfa) fruits [52], which could be because these fruits are relatively tolerant to drought stress [81]. With regard to less abundant organic acids, the two chili species previously mentioned showed different responses [43]. Under this deficit, quinic, shikimic, and fumaric acids decreased in the variety Chili-AS Root and increased in Naga Morich, while oxalic acid increased in Naga Morich but remained unaltered in Chili-AS Root [43], showing genotype-dependent changes within these organic acids.

#### 3.1.3. Amino Acids

Variations in metabolic responses regarding amino acid content have been observed in grape cultivars under treatments with decreased irrigation. At later developmental stages under the same stress severity, an increase in the total amino acid content was reported in the cultivar Tempranillo [25], while, in Cabernet Sauvignon grapes, no significant changes were observed, and, in the cultivar Shiraz, a decrease in amino acids was observed [48]. An increase in amino acids is a common response under drought stress due to nitrogen reallocation and an upregulation in protein catabolism [140]; thus, the observed reduction in Shiraz grapes is a less common phenomenon, which could be related to a decrease in carbon skeleton availability, likely due to impaired mitochondrial function under stress [48]. 

In addition, proline, which has an osmoprotective function [141], increased in both Tempranillo and Shiraz cultivars [25,48]. Remarkably, at early developmental stages, an increase in the phenylalanine content was observed in the two clones of the Tempranillo cultivar, which was accompanied by an observed decrease in anthocyanins, which could be explained by a reduction in phenylalanine ammonia lyase enzyme activity or amount in these fruits under these stress conditions [25].

#### 3.1.4. Fatty Acids

Oleic acid, which is important in olives, does not decrease under a water deficit; in fact, as irrigation increases, this compound decreases, as reported in the Cornicabra [58] and Arbequina [59] cultivars. By contrast, no change in the oleic and palmitic acids was observed in the cultivar Cobrançosa under the same stress [60]. On the other hand, polyunsaturated fatty acids, such as linoleic and linolenic acids, increase with irrigation [58,59]. However, these changes are not significant, and therefore they do not impact the final oil quality [59].

Phytoprostanes and phytofuranes, products of heat-treated polyunsaturated fatty acid oxidation, are bioactive compounds related to beneficial effects in both humans and plants [61]. In pistachios (*Pistacia vera* L.), phytoprostanes increased under moderate stress but decreased under severe stress, showing similar contents as fruits under control conditions [61]. This decrease under severe conditions could be explained by a decrease in these compounds when their concentration reaches a threshold as stress increases. The phytofurane total content was similar in plants under moderate stress compared with controls but decreased with severe stress [61]. 

In contrast, in almonds, the total content of phytoprostanes increased as stress increased, being higher under severe stress, while the total phytofuranes were higher under moderate stress than under severe stress [62]. In this sense, phytoprostanes and phytofuranes can be considered as potential osmotic stress biomarkers. While their increase under drought stress can be a consequence of enhanced ROS production resulting in the formation of lipid peroxidation products, their decrease under severe conditions can be caused by the oxidation of linolenic acid to jasmonic acid [61]. 

Therefore, the effects on the total content of these beneficial compounds depend on the species. Moreover, when individual analyses of individual compounds are performed, the obtained profiles depend on the severity of the stress; therefore, they can be used as markers to determine which kind of stress the plant has suffered [62]. For example, the most abundant phytoprostanes in pistachios increased, as did their total content, under moderate stress, while less abundant phytoprostanes were highest in plants under full irrigation regimes [61].

### 3.2. Impacts on Secondary Metabolism

#### 3.2.1. Polyphenols

Although water stress can cause a reduction in crop yield, fruits can increase their quality as secondary metabolites are produced [59]. For example, the total phenolic content increased in Tempranillo grapes [76] and in olive cultivars Cobrançosa [59] and Cornicabra [58]. This observed increase can be due to changes in enzyme activities, such as phenylalanine ammonia lyase, whose activity can be greatly increased under water stress [58]. However, although the total content increases, fruit quality can decline if relevant compounds decrease [59]. 

Therefore, individual analysis must be performed to determine fruit quality. For example, the phenols oleuropein and tyrosol, which greatly determine olive quality, change differently under water stress. Oleuropein, which influences oil stability and provides a bitter flavour to the fruit [63], increases in Picual [63] and Cobrançosa [59] cultivars under water stress, while hydroxytyrosol, produced from the hydrolysis of oleuropein and having a high antioxidant activity, decreases under this stress [63].

##### Cinnamic Acid

Cinnamic acid, produced from phenylalanine by the enzyme phenylalanine ammonia lyase, is the precursor of all compounds produced by the phenylpropanoid pathway [18,125]. Therefore, alterations in the concentration of this compound could imply that this pathway is being modified. In strawberries from the cultivar Camarosa with lower irrigation, a decrease in this compound was found, likely due to an increase in the production of phenylpropanoids to cope with the stress, thereby, activating the plant’s defences [46].

##### Anthocyanins

An increase in anthocyanins is commonly related to an increase in sugar, which promotes the expression of anthocyanin synthesis enzymes, such as *chalcone synthase* and *leucoanthocyanidin dioxygenase* [45]. An increase in these compounds in grapes causes an earlier end to veraison, as the maximum content is reached earlier [45]. Harvested red grapes that had been under water stress showed increased total anthocyanin content and, consequently, higher antioxidant activity [25,45,48,70,71]. However, as different cultivars present different anthocyanin profiles, the impacts of stress on individual anthocyanins also vary between cultivars [74]. 

In Shiraz grapes, glycosylated and acetylated forms changed more than in Cabernet Sauvignon, while acylated derivatives increased in Cabernet Sauvignon and not in Shiraz [48,71]. While delphinidin-3-*O*-glucoside, cyanidin-3-*O*-glucoside, and petunidin-3-*O*-glucoside increased under water deficit in Shiraz grapes, only petunidin-3-*O*-glucoside increased in Cabernet Sauvignon [48,70]. Additionally, the stage in which the stress is applied influences the modulation of the anthocyanin profile. Stress before veraison led to an increased anthocyanin content, except for malvidin and *p*-coumaroylated derivatives, which remained unaltered, while after veraison, the observed accumulation was particularly due to increases in malvidin and *p*-coumaroylated derivatives [71]. Thus, modulating irrigation and applying different strategies could lead to a desired anthocyanin profile in fruits.

Interestingly, plants have the potential to detect stress severity and to elaborate an appropriate response in each specific situation. In Crimson seedless grapes, a higher increase in anthocyanins was observed under irrigation deficit if the water was supplied by alternating between wet and dry root zones compared with plants under irrigation deficit with water supplied always to the same root zone [73]. In this sense, the perception of water stress appears to be enhanced compared with reduced deficit irrigation, and phenolic synthesis is upregulated as a defence mechanism. 

These experimental results suggest that water limitations during berry development may not be detrimental to fruit quality; in contrast, the bioactive composition of the fruit could show improvements without affecting the yield [73]. The effects are also stage dependent, as a late deficit increased the anthocyanin content in Tempranillo grapes, while it decreased with an early deficit [25]. In Chardonnay, which are white grapes and thus lack anthocyanin biosynthesis, no changes were observed in these compounds, thus to cope with the stress, they have to produce other types of compounds, such as flavonols, flavanols, and proanthocyanidins [70].

##### Flavonols

The flavonol content in fruits under drought stress differs between species and/or cultivars [74]. In Cabernet Sauvignon, Shiraz [48], and Tempranillo grapes [76], lower contents of these compounds were found [48], while, in Crimson seedless [73] and Sauvignon vert grapes [79] and in Cobrançosa olives [59], higher contents were detected. In fact, in Sauvignon vert grapes, the increase in kaempferol-3-*O*-glucoside, quercetin-3-*O*-glucoside, and quercetin-3-*O*-glucoronide was accompanied by an increase in the expression of *flavonol synthases* at later stages [79]. 

The way this stress is applied also influences the contents, as in Crimson seedless grapes, nonirrigated fruits presented higher contents of these compounds than reduced irrigated fruits [73]. In olives, no alterations were found in quercetin-3-*O*-glucoside, while its derivative quercetin-3-7′-di-O-glucoside increased under deficit irrigation, which can influence the health benefits to humans [59].

##### Flavanols

The effects of water stress on the flavanol content are cultivar dependent. At harvest, they were reduced in Cabernet Sauvignon [48] and Sauvignon vert [79] grapes, increased in Crimson seedless grapes [73], and were not affected in Shiraz grapes [48,71] when compared with control fruits. The type of applied stress also influences the flavanol concentration, such as in Crimson seedless grapes, in which the observed increase in flavanols was higher in non-irrigated plants and reduced in plants with irrigation alternating between wet and dry zones, while with only reduced irrigation the increase was not significant compared with control fruits [73]. 

Remarkably, although stress does not affect the flavanol total content in a cultivar, such as in Shiraz grapes, its extraction can be easier under stress, and therefore its bioavailability increases, likely due to a reduction in the interactions with other molecules [71]. Strikingly, combining transcriptomic and metabolomic analyses, Savoi et al. [79] observed an upregulation in the expression of key structural genes for flavonol and flavanol synthesis under water deficit at late stages of berry development, while no increase in these metabolites was detected, except for proanthocyanidin procyanidin B1, whose content was higher at harvest. The authors suggested competition between phenylpropanoid and flavonoid enzymes for precursors, with the first ones being more efficient in driving carbon flux toward the synthesis of benzoic and cinnamic acid derivatives, such as gallic acid [79].

##### Stilbenes

Resveratol, likely the best known stilbenoid for its presence in grapes and wine, has a role as an antioxidant and chemoprotectant. In grapewine, it has been shown that the level of resveratrol was found to be cultivar-dependent under drought stress [16,73,74,84]. In fact, in the cultivars Chardonnay and Cabernet Sauvignon, similar stilbene concentrations were reported in well-watered plants; however, under deficit irrigation conditions, trans-piceid, the glycosylated form of trans-resveratol, increased only in Cabernet Sauvignon grapes, which is consistent with the transcriptomic analysis performed, showing an upregulation of stilbene synthase [84]. The stress duration also influenced its content, and if the stress lasted longer, a higher increase was reported in Hutai No. 8 grapes [16]. Finally, the implementation of irrigation strategies can result in grapes with higher resveratol, as was reported for grapes with reduced irrigation but alternating between wet and dry root zones [73].

#### 3.2.2. Terpenoids

##### Carotenoids

Commonly, no significant changes have been observed in the total carotenoid content in a wide variety of crops suffering drought stress, although slight increases, as reported for some tomato cultivars [42] and mangoes from the cultivar Osteen [81], or decreases, as observed in mangoes from the Cogshall cultivar [82], have been detected. In fact, the modulation of carotenoid genes under deficit irrigation in Sauvignon vert grapes was much less intense than the modulation of phenylpropanoid genes [79]. 

However, if individual analyses were performed, alterations of single compounds were detected; violaxanthin, beta-carotene, and antheraxanthin increased in *M. indica* cv. Osteen [81], antheraxanthin increased in Chardonnay grapes before and after veraison, and violaxanthin decreased in Cabernet Sauvignon grapes [70]. On the other hand, carotenoid degradation during berry ripening was found to be enhanced in Sauvignon vert grapes under water stress, resulting in fruits with a lower content of these compounds [79]. Surprisingly, although carotenoid degradation increased during water stress, no effect was observed on the content of C_13_-norisoprenoid/apocarotenoid volatiles, which originate from carotenoid cleavage.

##### Monoterpene Volatiles

In contrast, water deficit positively impacts the monoterpene content, which is an important aroma volatile in some white grape cultivars. The monoterpene increase was paralleled by the induction of key structural genes in the MEP pathway [79]. Interestingly, the authors also observed an enrichment in drought-associated elements in the promoter regions of the terpenoid genes that were upregulated under drought stress, suggesting direct modulation at the transcriptional level [79].

##### Triterpenes

Triterpenes are found at high concentrations in olive fruits, where they determine fruit quality together with phenolic compounds, as they present beneficial properties in humans due to their antioxidant activity [63]. The contents of oleanoic and maslinic acids, the triterpenes found in olive fruits, are reduced in nonirrigated plants, therefore, decreasing the fruit quality [63].

#### 3.2.3. Vitamin C or Ascorbate

Stress tolerance increases antioxidant molecules, such as ascorbic acid, which acts as a substrate for enzymatic detoxification [11]. Thus, an increase in this molecule has been associated with tolerance against drought stress [11], which has been observed in Shiraz grapes [48] and in different chili species [11]. However, in the chili cultivars Chili-AS Rot and Naga Morich, a decrease was observed. Again, a stress duration dependence occurred in these chilies that increased the ascorbic acid content under irrigation deficit. Compared with control fruits, the cultivars Jolokian and Puerto Rican had a decrease in this compound under drought stress that lasted longer, while the Bird’s Eye cultivar appeared more tolerant to this stress; although the ascorbic acid concentration decreased when the stress lasted longer, it was still higher than the control plants [11].

#### 3.2.4. Capsaicin and Dihydrocapsaicin

Capsaicin and dihydrocapsaicin are molecules with antioxidant activities and are responsible for the characteristic pungency in chilies [11]. As the principal interest in these fruits is this characteristic pungency [43], changes in the concentration of these substances greatly determine consumer acceptance. Interestingly, Kopta et al. [11] observed an increase in pungency under drought conditions. However, the effects of drought stress on these compounds are cultivar dependent [11]; cultivars that typically show high pungency and consequently have higher concentrations of these compounds do not show significant alterations or may even decrease under this stress, as reported for *C. chinense* var. Naga Morich [43]. 

In addition, the activity of capsaicin synthase was reduced under water stress in highly pungent cultivars [142]. More sensitive cultivars, usually characterized by low pungency, highly upregulate the synthesis of these compounds, such as in *C. chinense* Jacq. Jolokia [11]. Thus, considering that the response is cultivar-dependent, it is also determined by stress duration, and for cultivars *C. anuum* Bird’s Eye and *C. chinense* Jolokia and Puerto Rican, an initial increase was observed when the stress lasted 21 days but decreased when it lasted longer [11].

## 4. Irradiance

**Figure 3:**
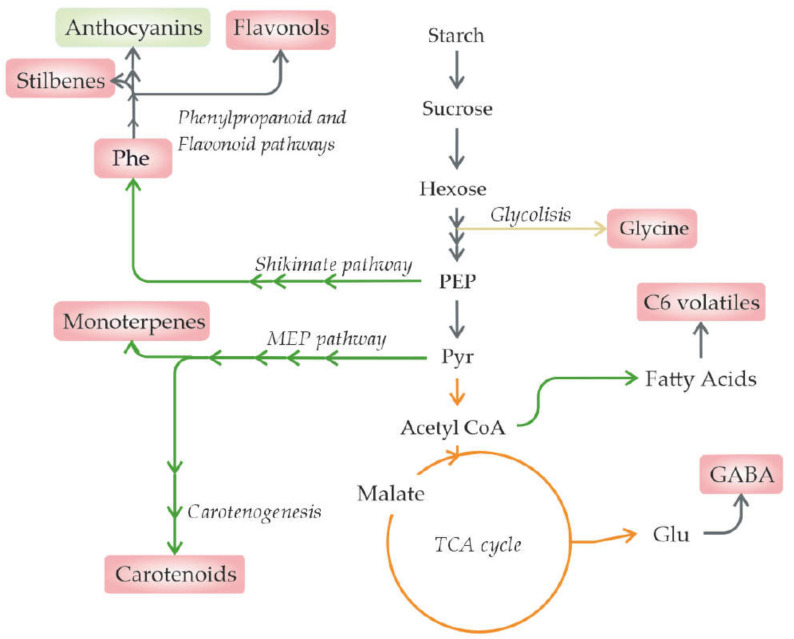
General effects of an increase of irradiance in fruit metabolism. Red: increase; blue: decrease; and green: changes in its patterns. Arrows: grey: cytosol; orange: mitochondria; green: plastids; and yellow: peroxisome.

In fruits, solar irradiance principally influences fruit pigments. Mainly, the information available regarding this effect is related to grapes, as its pigment content greatly influences the final wine colour and, thus, has an important socioeconomic impact [75]. As irradiance produces metabolic changes (Figure 3), different strategies are being developed and are currently being implemented to mitigate the effects from other stresses, such as temperature increases, aiming to modulate these metabolites as desired [19]. These strategies include leaf removal, the use of nets that decrease the total irradiation, and selective nets that filter specific wavelengths [69,75,77].

### 4.1. Impacts on Primary Metabolism

Although the effects of solar irradiance on the primary metabolism are less pronounced than the observed changes produced in specialized metabolisms, some variations are still observed. This generally unaffected metabolic composition is consistent with the fact that the expression of genes associated with growth and the primary metabolism is mainly unaltered under light exposure in Sauvignon Blanc grapes [54].

#### 4.1.1. Sugars

Relating to sugars, no significant changes were observed in Sauvignon blanc grapes after leaf removal at different developmental stages, although a slight increase was detected in grapes directly exposed to solar irradiance, as berries are photosynthetically active in the early developmental stages and, therefore, can produce these compounds when th4 leaves have been removed [44]. Additionally, as the light incidence depends on the position of the fruit, individual analyses taking into account the side of the canopy have been performed. At harvest, the glucose, fructose, and sucrose contents were relatively uniform across the canopy in Cabernet Sauvignon grapes [19]. Therefore, other environmental factors influence the sugar content more than solar irradiance.

#### 4.1.2. Organic Acids

The influence of leaf removal on organic acids is more heterogeneous compared with the lack of variations in sugars; for example, in Sauvignon blanc grapes, an increase in tartaric acid and a decrease in malic acid were observed in exposed berries [44]. This was also observed in Cabernet Sauvignon grapes when studying the effect of canopy side, obtaining an increase in tartaric acid in highly exposed grapes and a decrease in malic acid in these grapes, having higher concentration in less exposed grapes in the canopy [19]. However, these results could be due to the effect of an increase in temperature produced by the higher exposure instead of a result of the exposure [44].

#### 4.1.3. Amino Acids

Amino acids can act as precursors of many compounds and are usually related to stress tolerance; in some cases, they can be used as markers in fruits that have been under solar irradiance [54]. Upregulation of enzymes involved in amino acid catabolic reactions under light stress may indicate limitations in berry energetic resources, which result from the activation of photoprotective mechanisms; indeed, amino acid degradation may provide metabolic energy, allowing the maintenance of primary metabolism under stressful environmental conditions [54]. In fact, after veraison, a decrease in the total amino acid content was observed in Sauvignon Blanc exposed to light, possibly indicative of the energetic cost of the light protective mechanisms. 

However, specific and dependent-stage patterns have been observed for some amino acids; glycine increased in all increased light exposure treatments; aspartate, asparagine, glutamate, glutamine, alanine, arginine, cysteine, phenylalanine, and tryptophan decreased at veraison; and while at ripeness, GABA, methionine, proline, and valine increased with increased light exposure and arginine, asparagine, phenylalanine, and tryptophan were found at lower concentrations. In particular, GABA and proline increases are suggestive of abiotic stress symptoms; possibly, the oxidative homeostasis of the ripe berry cannot be maintained for a longer period of time, although no visible difference could be detected between light- and shade-exposed fruits. 

The increase in glycine in unmature berries can be associated with its role in photorespiration, a mechanism by which photosynthetic machinery is protected from light stress. To outline the function of this amino acid in the protection of photosynthetic machinery, the glycine content difference between light- and shade-exposed berries decreases as the photosynthesis rate decreases through ripening [54]. Interestingly, glycine is also considered the rate-limiting metabolite in the synthesis of glutathione, an important antioxidant [54]. 

Remarkably, an upregulation of genes involved in the tryptophan, phenylalanine, and tyrosine catabolism was reported, while no significant changes were observed in genes involved in their biosynthesis; therefore, they could be implicated in the synthesis of secondary metabolites; however, they could also be used as an energy source to maintain plant growth under stressful conditions [54]. In Cabernet Sauvignon grapes, increases in leucine, valine, alanine, serine, and GABA were observed as light increased, while proline, beta-alanine, and threonine did not show significant changes [19]. In this way, changes in amino acid patterns can be used as irradiance stress markers.

### 4.2. Impacts on Secondary Metabolism

The effects of solar irradiance on secondary metabolism are more pronounced, and commonly, significant variations are observed. Typically, pigments are the compounds most influenced by solar irradiance, as they act as UV protectors [75]. In this way, flavonoids and carotenoids are the most influenced by light, as the enzymes involved in their metabolism are regulated by this stimulus [64,83].

#### 4.2.1. Polyphenols

##### Flavonoids

The presence of fruit photoreceptors has been shown to induce flavonoid synthesis (reviewed in [143]). In particular, light and UV radiation upregulate the expression of genes encoding *R2R3 MYB,* and *bHLH* transcription factors, which, in turn, activate the structural genes in the phenylpropanoid and flavonoid pathways [19,54,77].

##### Anthocyanins

The anthocyanin content is influenced by light exposure, although it seems to present a higher dependence on temperature changes [19]. Different outcomes have been observed in different cultivars using several treatments. An enhancement of anthocyanin accumulation has been reported in Pinot Noir cultivars [65,68], although differences between locations were observed, as in one of the locations, plants had smaller leaves; thus, light penetrated more in nontreated canopies, showing lower differences between treated and nontreated plants [68]. In contrast, a higher content was detected in Cabernet Sauvignon grapes under shading treatment [75]. 

In Merlot grapes, an increase in the total anthocyanin content was observed when leaf removal was performed at the prebloom stage. This increase could be explained because, if the removal was performed at postfruit set, the berry skin mass and vacuolar storage would have decreased, consequently decreasing the anthocyanin content. On the other hand, the same experiment was performed the following year, and the opposite result was obtained, presenting higher anthocyanin after postfruit set removal, which could be explained because, during that year, a decrease in the yield occurred, and the source/sink ratio increased, promoting anthocyanin accumulation [67].

However, generally, no significant changes in the total anthocyanin content have been reported [66,69], the anthocyanin profile is commonly influenced by different light treatments, and its effects depend on the studied cultivar. For example, in Nero di Troia grapes, an increase in delphinidin-3-glucoside, cyanidin-3-glucoside, petunidin-3-glucoside, peonidin-3-glucoside, and peonidin-3-coumaroylglucoside occurred after removing all leaves from the western side of the fruit zone. 

On the other hand, in fruits under higher irradiance and temperature due to the removal of 75% of the leaves in eastern and eastern/western situations, delphinidin-3-glucoside and malvidin-3-p-coumaroylglucoside increased in the eastern removal, and petunidin-3-glucoside, peonidin-3-glucoside, and peonidin-3-p-coumaroylglucoside increased in the eastern/western removal [69]. For all treatments, a decrease in malvidin-3-glucoside was reported [69], as was also observed in Cabernet Sauvignon grapes, which presented a higher content of this compound in less exposed grapes and decreased with higher exposure to solar irradiance [19,75]. 

In this cultivar, an increase in cyanidin-3-glucoside was reported in highly irradiated fruits and decreased as shading increased [19,75]. The moment of fruit development is when removal occurs, and other environmental conditions also influence the changes in the anthocyanin profile; in Pinot Noir grapes in two different locations, a defoliation treatment was performed at two developmental stages: berry set and veraison. In one of the locations, delphinidin-3-glucoside, petunidin-3-glucoside, and malvidin-3-glucoside were higher after leaf removal at berry set, while peonidin-3-glucoside content was higher in untreated fruits; in the other location, only cyanidin-3-glucoside and peonidin-3-glucoside increased in fruits treated at berry set [68].

Additionally, the effects of changes in the light spectra can be observed, as not all grapes receive the same amount of each type of light. In this way, small but significant changes were observed in Cabernet Sauvignon grapes, which decreased delphinidin-3-glucoside and petunidin-3-glucoside in fruits under 30% blue shading [75].

Therefore, different outcomes are observed depending on the studied cultivar and the treatment applied. The main interest in these changes is the wine colour that will be obtained, as grapes with higher trihydroxylated forms (delphinidin, petunidin, and malvidin) present a more purple colour than those with higher dihydroxylated forms (cyanidin and peonidin). In the cultivars Nero d’Avola and Sangiovese, higher contents of dihydroxylated forms were obtained after leaf removal treatment [66]. Additionally, these changes could be modulated by applying the treatment at different developmental stages, as in Pinot Noir grapes, an increase in trihydroxylated forms occurred when leaf removal started at bloom. 

However, in grapes of this cultivar in another location, when leaf removal started at bloom, fruits presented higher contents in dihydroxylated forms, but when the treatment started at grain-pea berry size, higher trihydroxylated anthocyanins were found. Interestingly, the enzymes responsible for di- and trisubstituted anthocyanins, i.e., F3′H and F3′5′H, respectively, were shown to respond to both light and temperature [66,110,144]. Interestingly, *F3′5′H* expression was downregulated in Cabernet Sauvignon-exposed berries (leaf removal at veraison), coinciding with the higher dehydroxylated/trihydroxylated flavonoid ratios observed under high light exposure [78]. Additionally, wine anthocyanin composition can be influenced by irradiation, as it can affect the extraction and its stability during the process [69,75].

##### Flavonols

Flavonols, which are important molecules because they act as copigments influencing anthocyanin stability [69] and as sunscreen metabolites preventing ROS production, tend to increase in more exposed grapes and decrease (to negligibly low levels) in less exposed grapes [19,66,67,75,77,78,80], which is in accordance with an upregulation of the expression of genes related to flavonol biosynthesis [77,78]. The induction of flavonol biosynthesis is commonly related to decreases in anthocyanin concentrations, as the precursors utilize the flavonol synthesis pathway [19]. Irradiance dominates flavonol content modulation [66], although it is influenced by other factors; therefore, the obtained degree of change usually varies between seasons [78].

During development, changes in individual flavonols can be detected; however, these differences do not always persist until harvest. For example, in Cabernet Sauvignon grapes, higher levels of quercetin-3-glucoside and kaempferol-3-glucoside were obtained under solar irradiance at veraison; however, at harvest, only kaempferol-3-glucoside presented significant differences between treatments [75]. 

The location of the canopy also affects the flavonol profile modification, as in Cabernet Sauvignon grapes located on the east side, a decrease in myrecitin-3-glucoside, myrecitin-3-glucoronide, quercetin-3-glucoside, kaempferol-3-glucoside, and kaempferol-3-glucoronide was observed under shading conditions, while, in west-located grapes, the decrease was only observed in myricetin-3-glucoside, quercetin-3-glucoside, kaempferol-3-glucoside, and kaempferol-3-glucoronide [19].

As in anthocyanins, light spectra influence flavonol profiles. As in Cabernet Sauvignon grapes, when blue light was filtrated, a decrease in kaempferol-3-glucoronide was detected [75]. UV-B light influences flavonol synthesis, as a lower content has been reported in grapes under UV-B attenuated conditions [77], which is consistent with transcriptomic analysis, where an upregulation of MYB transcription factors that activate flavonol biosynthetic genes, such as chalcone synthases and flavonol synthases, was observed with UV-B radiation [77].

##### Flavanols

Flavanols are the most abundant flavonoids in grapes, followed by anthocyanins and flavonols [78]. Commonly, no significant changes in these compounds have been reported under different light treatments, as reported in Nero di Troia [69] and Pinot Noir grapes [80]. In Cabernet Sauvignon, flavanols increased in less exposed grapes and decreased in the highly exposed grapes at veraison; however, these differences were not significant at harvest [19,75].

##### Stilbenes

Stilbenes have been described to accumulate in grapevines in response to UV-C irradiation treatments [145]. While no correlation was found between solar irradiance and stilbene content in Cabernet Sauvignon, their levels were significantly higher in exposed clusters compared with in shaded clusters [19]. Interestingly, as in stilbene synthase and chalcone synthase, which are involved in flavonoid formation and compete for the same precursors, a shift in metabolic coordination is observed under light exposure. In particular, the correlation between stilbenes and flavonols shifts from negative (in shaded clusters) to positive (in exposed clusters), with a concomitant increase in these two classes of polyphenols and a decrease in anthocyanins under high solar irradiance. Together, these data suggest a differential repartitioning of carbon precursors under light stress [19].

#### 4.2.2. Terpenoids

##### Carotenoids

Carotenoids participate in plant acclimation to high radiation exposure, as they are related to photosynthesis, mitigating light excess [77]; therefore, an increase in these compounds is expected under light stress conditions. Young et al. [44] studied the effects on leaf removal in the bunch zone in the Sauvignon Blanc cultivar using a field-omic workflow. Therefore, light exposure and, to a lesser extent, temperature increased, with a major impact on the carotenoid metabolism in the early stages of berry development, when the fruit was still photosynthetically active. Interestingly, they demonstrated that the carotenoid metabolism was regulated at both the transcriptional and metabolite levels as part of plant acclimation responses. 

Most noteworthy, an increase in photoprotective xanthophylls and a decrease in the ratios of β-carotene-lutein and lutein epoxide-lutein were observed in light-exposed, unmature berries. Notably, even if major changes occurred in the early stages of berry development, they also impacted the final composition of the ripe fruit. Indeed, the increased carotenoid pool resulted in enhanced levels of apocarotenoid volatiles in the later stages of fruit development, as the major xanthophyll pool in exposed berries may possibly serve as substrates for their synthesis [44,54,77]. 

However, in a further study combining high and low light exposure with UV attenuation, Joubert et al. [77] observed that the amplitude of the xanthophyll and lutein epoxide cycles, which are photoprotective mechanisms, was dependent not only on solar irradiance but also on UV-B exposure; as likely, without UV-B radiation, fewer carotenoids are needed to mitigate light stress. On the other hand, in tomatoes of the cultivar Ailsa Craig, the effect of UV-B shielding led to an increase in total carotenoids in the skin of the mature green and breaker stages but then decreased in the red ripe stage. In the flesh, no significant changes were observed under UV-B shielding conditions. 

Notably, UV-B depletion negatively affected ethylene synthesis in the Aisla Craig cultivar, implying an important role for solar radiation in the tomato-ripening process. Using *rin* and *nor* mutants, which impaired ethylene production, significant changes in carotenoid contents were observed, which elucidates that, although ethylene controls carotenoid concentration, its accumulation is also influenced by UV-B shielding, and therefore, there is an additional pathway involved in carotenoid production that is not controlled by ethylene [83]. An increase in the total carotene content was also detected in cherry tomatoes under increased irradiance during ripening compared with fruits that were kept in the dark, as red light modulates the carotenogenetic process by modulating enzymes, such as phytoene synthase [64].

Regarding individual carotenoids, lycopene, beta-carotene, and their precursors phytoene and phytofluene increased with higher irradiance exposure in cherry tomatoes [64] and decreased in Ailsa Craig tomatoes under UV-B shielding [83].

##### Monoterpene Volatiles

Furthermore, monoterpene volatiles also generally increased in exposed Sauvignon Blanc berries, as previously observed in Malbec cultivars with enhanced UV-B radiation [44,77,146]. Monoterpene emissions increased in response to both biotic and abiotic stress [147]; however, their exact ecological/physiological roles remain elusive. Young et al. [44] suggested that they may play roles in membrane stability (due to their lipophilic nature) and photoprotection, compensating for carotenoid depletion in exposed berries.

#### 4.2.3. Volatile Compounds

C6 volatiles, such as hexanal and *trans*-2-hexenal, increased in Sauvignon Blanc grapes under a high light microclimate compared with high light with UV-B attenuation. These results suggest that UV light is somehow involved in the polyunsaturated fatty acid metabolism [77]. Interestingly, straight-chain aldehydes, such as 2-heptanal and *trans*-2,4-heptadienal, and the ketone 1-octen-3-one, showed the same tendency.

## 5. Conclusions (Future Perspectives for Breeding under Challenging Environmental Conditions)

Future climate predictions are associated with shifting environmental factors, such as increasing temperature, changes in precipitation patterns, and extreme weather conditions that affect plant growth. These changes will not only affect the physiological processes of plants that lead to yield loss but also they will influence the fruit quality, which could have a high socio-economic impact. Therefore, there is an urgent need to improve our understanding of plant responses to these environmental factors as this will help in developing tolerant genotypes and in anticipating the impacts on agriculture.

Although stress tolerance mechanisms are highly complex, advances in functional genomics, including transcriptomics, proteomics, and metabolomics together with other high-throughput phenotyping platforms, plant modeling, and data processing, as we presented in this review, have provided new insights for elucidating the underlying novel genomic region(s), candidate gene(s), gene networks, novel proteins and metabolites, and other signaling molecules contributing to stress tolerance. However, there is a need to explore the germplasm strategically, including wild relatives, landraces, and gene pools to discover new sources of variations that could further identify new adaptive traits contributing to plant adaptations to new environments to develop alternative and competitive strategies that would be a great help to mitigate climate change impacts [148].

## Figures and Tables

**Table 1:** Metabolomic platforms used in different metabolite determinations.

Metabolite	Platform	Fruit	Reference
Sugars	HPLC-PDA-MS	*Ribes nigrum* L.	[35]
	HPLC-PAD	*Vitis vinifera* L. cv. Cabernet Sauvignon	[36]
	HPLC-PDA/RID	*Fragaria x ananassa* Duch., cvs. Festival, Fortuna and Rubygem	[37]
	HPLC	*Vitis vinifera* L.	[38]
		*Solanum lycopersium* L. cv. Money-maker	[39]
		*Ribes nigrum* L.	[10]
		*Vitis vinifera* L.	[40]
		*Lycopersicum esculetum* Miller cv. Jinpeng 11	[41]
		*Solanum lycopersicum* L. cvs. Ferum, LA0147, Levovil, Stupicke Polni, Rane, Cervil, Criollo, La1420 and Plovdiv)	[42]
		*Capsicum annuum* L. var Chili-AS Rot and *Capsicum chinense* Jacq var Naga Morich	[43]
	Reverse-phase HPLC	*Vitis vinifera* L. cv. Sauvignon blanc	[44]
	LC/MS/MS	*Vitis vinifera* L. cv. Cabernet Sauvignon	[45]
	LC-MS	*Fragaria x. ananassa* Duch. Camarosa	[46]
	GC-MS GC ULTRA DSQII MS	*Vitis vinifera* L. cv. Gamay Red	[47]
	GC-MS	*Solanum lycopersicum* L. cv. Ailsa Craig	[13]
		*Vitis vinifera* L. cv. Shiraz and Cabernet Sauvignon	[48]
		*Actinia deliciosa* cv. Hayward	[49]
		*Vitis vinifera* cv. Cabernet Sauvignon	[19]
Organic acids	HPLC-PDA/RDI	*Fragaria x ananassa* Duch. cvs. Festival, Fortuna and Rubygem	[37]
	HPLC	*Vitis vinifera* L.	[38]
		*Solanum lycopersicum* L. cv. Money-maker	[39]
		*Ribes nigrum* L.	[10]
		*Vitis vinifera* L.	[40]
		*Fragaria x. ananassa* Duch.	[50]
		Tropical fruits	[51]
		*Solanum lycopersicum* L. cvs. Ferum, LA0147, Levovil, Stupicke Polni Rane, Cervil, Criollo, La1420 and Plovdiv)	[42]
		*Capsicum annuum* L. var Chili-AS Rot and *Capsicum chinense* Jacq var Naga Morich	[43]
		*Magnifera indica* L. cv. Lirfa	[52]
	Reverse-phase HPLC	*Vitis vinifera* L. cv. Sauvignon blanc	[44]
	LC/MS/MS	*Vitis vinifera* L. cv. Cabernet Sauvignon	[45]
	GC-MS	*Vitis vinifera* L. cvs. Shiraz and Cabernet Sauvignon	[48]
		*Vitis vinifera* L. cv. Cabernet Sauvignon	[19]
Amino acids	HPLC	*Vitis vinifera* L. cv. Cabernet Sauvignon	[53]
		*Vitis vinifera* L. cv. Sauvignon blanc	[54]
	HPLC-PDA-MS	*Ribes nigrum* L.	[35]
	UHPLC-FLD 3000 fluorescence detector	*Vitis vinifera* L. cv. Tempranillo	[25]
	GC-MS	*Vitis vinifera* L. cv. Cabernet Sauvignon	[19]
		*Vitis vinifera* L. cvs. Shiraz and Cabernet Sauvignon	[48]
		*Vitis vinifera* L. cv. Gamay Red	[47]
		*Magnifera indica* L. cv. Chaunsa white	[55]
Fatty acids	GC	*Olea europaea* L. cv. Arauco	[56]
			[57]
	GC-flame ionization detector	*Olea europaea* L. cvs. Zelmati, Cornicabra, Cobrançosa	[24,58,59]
	GC-MS	*Solanum lycopersicum* L. cv. Ailsa Craig	[13]
		*Olea europaea* cv. Arbequina	[60]
Phytoprostanes and phytofuranes	UHPLC-QqQ-MS/MS	*Pistacia vera* L. cv. Kerman	[61]
		*Prunus dulcis* Mill. cv. Vairo	[62]
Phenolics	HPLC-UV/Vis and HPLC-MS/MS	*Olea europaea* cv. Picual	[63]
	HPLC	*Solanum lycopersicum* L. cv. Cervil	[64]
		*Fragaria x ananassa*, cvs. Festival, Fortuna and Rubygem	[37]
	HPLC DAD UV-vis	*Solanum lycopersicum* L. cv. Money-maker	[39]
	HPLC-UV detector	*Olea europaea* L. cv. Cornicabra	[58]
	HPLC-UV-visible photodiode detector	*Olea europaea* L. cv. Cobrançosa	[59]
	LC-MS UPLC-QTOF-MS	*Vitis vinifera* L. cv. Gamay Red	[47]
Anthocyanins	HPLC	*Vitis vinifera* L. cv. Pinot noir	[65]
		*Vitis vinifera* L. cvs. Cabernet Sauvignon, Nero d’Avola, Raboso Ppiave, Sangiovese	[66]
		*Vitis vinifera* L. cv. Merlot	[67]
		*Vitis vinifera* L. cv. Cabernet Sauvignon	[53]
	HPLC UV-vis	*Vitis vinifera* L. cv. Pinot noir	[68]
	HPLC with photo-diode array detector	*Prunus salicina* L. cv. Red Beauty	[28]
	HPLC–DAD–ESI-MS/MS analysis	*Vitis vinifera* L. cv. Nero di Troia	[69]
	HPLC-PDA-MS	*Ribes nigrum* L.	[35]
	HPLC-UV visible detector	*Vitis vinifera* L. cvs. Cabernet Sauvignon and Chardonnay	[70]
		*Vitis vinifera* L. cv. Shiraz	[71]
		*Vitis vinifera* L. cv. Cabernet Sauvignon	[72]
	UHPLC-DAD-3000 diode	*Vitis vinifera* L. cv. Tempranillo	[25]
	UPLC LC-30AD	*Vitis vinifera* L. cv. Crimson Seedless	[73]
	UPLC QTOF-MS	*Vitis vinifera* L. cv. Cabernet Sauvignon	[19]
	UPLC-MS	*Vitis vinifera* L. cvs. Shiraz and Cabernet Sauvignon	[48]
	UPLC-QqQ-MS/MS	*Vitis vinifera* L. (93 cultivars)	[74]
	UPLC-QTOF-MS	*Vitis vinifera* L. cv. Cabernet Sauvignon	[75]
Flavonols	UPLC–(ESI−)–MS/MS	*Vitis vinifera* L. cv. Tempranillo	[76]
		HPLC	*Vitis vinifera* L. cvs. Cabernet Sauvignon, Nero d’Avola, Raboso Piave, Sangiovese	[66]
			*Vitis vinifera* L. cv. Sauvignon blanc	[77]
HPLC–DAD–ESI-MS/MS analysis		*Vitis vinifera* L. cv. Nero di Troia	[69]
HPLC-MSD trap VL		*Vitis vinifera* L. cv. Cabernet Sauvignon	[78]
HPLC-PDA-MS		*Ribes nigrum* L.	[35]
UHPLC-UV-vis		*Vitis vinifera* L. cv. Tempranillo	[25]
UPLC LC-30AD		*Vitis vinifera* L. cv. Crimson Seedless	[73]
UPLC QTOF-MS		*Vitis vinifera* L. cv. Cabernet Sauvignon	[19]
		*Vitis vinifera* L. cv. Cabernet Sauvignon	[75]
UPLC-MS		*Vitis vinifera* L. cv. Cabernet Sauvignon	[48]
UPLC-QqQ-MS/MS		*Vitis vinifera* L. (93 cultivars)	[74]
UPLC-triple-quadrupole MS		*Vitis vinifera* L. cv. Sauvignon vert	[79]
Flavanols	HPLC	*Vitis vinifera* L. cv. Pinot noir	[80]
	HPLC–DAD–ESI-MS/MS analysis	*Vitis vinifera* L. cv. Nero di Troia	[69]
	HPLC-MSD trap VL	*Vitis vinifera* L. cv. Cabernet Sauvignon	[78]
	HPLC-UV-vis	*Vitis vinifera* L. cv. Shiraz	[71]
	LC-MS	*Vitis vinifera* L. cv. Gamay Red	[47]
	UPLC LC-30AD	*Vitis vinifera* L. cv. Crimson Seedless	[73]
	UPLC-MS	*Vitis vinifera* L. cvs. Shiraz and Cabernet Sauvignon	[48]
	UPLC-QTOF-MS	*Vitis vinifera* L. cv. Cabernet Sauvignon	[75]
	UPLC-triple-quadrupole MS	*Vitis vinifera* L. cv. Sauvignon vert	[79]
Carotenoids	HPLC	*Solanum lycopersicum* L. cvs. Ferum, LA0147, Levovil, Stupicke Polni Rane, Cervil, Criollo, La1420 and Plovdiv)	[42]
		*Magnifera indica* L. cv Osteen	[81]
	HPLC DAD UV-vis	*Solanum lycopersium* L. cv. Money-maker	[39]
		*Solanum lycopersicum* L. cv. Cervil	[64]
		*Magnifera indica* L. cv Cogshall	[82]
		*Solanum lycopersicum* L. cv. Cervil	[64]
	HPLC-UV/vis	*Solanum lycopersicum* L. cv. Ailsa Craig	[83]
		*Vitis vinifera* L. cvs. Cabernet Sauvignon and Chardonnay	[70]
		*Solanum lycopersium* L. cv. Velasco	[27]
	UPLC	*Vitis vinifera* L. cv. Sauvignon vert	[79]
		*Vitis vinifera* L. cv. Sauvignon blanc	[77]
	UPLC-PDA	*Solanum lycopersicum* L. cv. Ailsa Craig	[13]
Vitamin C	GC-MS	*Vitis vinifera* L. cvs. Shiraz and Cabernet Sauvignon	[48]
	HPLC	*Fragaria x ananassa* Duch. cvs Festival, Fortuna and Rubygem	[37]
		*Ribes nigrum* L.	[10]
	HPLC-PDA-MS	*Ribes nigrum* L.	[35]
	HPLC-UV-vid detector	*Capsicum chinense* Jacq. Jolokia and Puerto Rican, C. annuum Bird’s eye and *C. baccatum* L. cv. Aji Lemon Drop	[11]
	LC-MS	*Solanum lycopersium* L. cv. Velasco	[27]
Vitamin E	GC-MS	*Solanum lycopersicum* L. cv. Ailsa Craig	[13]
Volatiles	GC-MS	*Solanum lycopersicum* L. cv. Ailsa Craig	[13]
Capsaicin	HPLC-DAD detector	*Capsicum annuum* L. var Chili-AS Rot and *Capsicum chinense* Jacq. var Naga Morich	[43]
	HPLC-UV-vid detector	*Capsicum chinense* Jacq. Jolokia and Puerto Rican, *C. annuum* Bird’s eye and *C. baccatum* Aji Lemon Drop	[11]
Resveratrol	HPL	*Vitis vinifera* L. cv. Hutai No.8	[16]
		*Vitis vinifera* L. cv. Cabernet Sauvignon and Chardonnay	[84]
	UPLC LC-30AD	*Vitis vinifera* L. cv. Crimson Seedless	[73]
	UPLC-QqQ-MS/MS	*Vitis vinifera* L. (93 cultivars)	[74]

DAD: diode-array detection; ESI: electrospray ionization; ESI-: electrospray ionization in negative mode; FID: flame ionization detector; FLD: fluorescence detector; HPLC: high-performance liquid chromatography; PAD: pulsed amperometric detector; PDA: photo diode array; QqQ: targeted quadrupole; QTOF: quadrupole time-of-flight; RID: refractive index detector; UHPLC: ultra-high-performance liquid chromatography; UPLC: ultra-performance liquid chromatography; and UV/Vis: ultraviolet/visible spectroscopy.
